# Correlation of thyroid dysfunction and cognitive impairments induced by subcortical ischemic vascular disease

**DOI:** 10.1002/brb3.452

**Published:** 2016-03-14

**Authors:** Zongsheng Chen, Xianfa Liang, Chunxiu Zhang, Jinling Wang, Gaiping Chen, Hong Zhang, Zhongwu Sun

**Affiliations:** ^1^ Department of NeurologyThe First Affiliated Hospital of Anhui Medical University218 Jixi Road230022HefeiAnhuiChina; ^2^ Department of NeurologyTongling No. 4 People Hospital98 Yangjiashan Road244000TonglingAnhuiChina

**Keywords:** Mild cognitive impairment, MMSE, subcortical ischemic vascular disease, thyroid dysfunction, thyroid hormone

## Abstract

**Background:**

To date, the relationship between thyroid dysfunction and subcortical ischemic vascular disease (SIVD)‐induced cognitive impairments still remains elusive.

**Methods:**

Cognitive performances were examined in 215 participants, including 54 healthy participants, 52 SIVD patients with no dementia (SIVDND), 55 patients with mild cognitive impairment (SVMCI), and 54 patients with vascular dementia (VD). Serum thyroid‐stimulating hormone (TSH), total triiodothyronine (TT3), free triiodothyronine (FT3), total thyroxine (TT4) and free thyroxine (FT4), thyroglobulin antibody (TGA), and antithyroid peroxidase antibody (TPO‐Abs) were quantified by radioimmunoassay or ELISA.

**Results:**

A close correlation between thyroid status and cognitive dysfunction in SIVD was observed. Serum TT3 and FT3 levels decreased, whereas serum TSH level increased, with the decline in cognitive functions. Furthermore, TT3 levels showed a positive correlation, whereas TSH level showed a negative correlation, with the Mini‐Mental State Examination (MMSE) scores. Our results suggested that thyroid function was associated with cognitive impairments induced by SIVD. Also, thyroid function and thyroid hormone level could be a risk factor in the development of SIVD. Serum TT3 and TSH levels might also be used as biomarkers for cognitive dysfunction.

**Conclusions:**

These findings might contribute to a more accurate clinical diagnosis and differentiation among normal controls, SIVDND, SVMCI, and VD patients, in order to develop appropriate intervention approaches for SIVD therapeutic treatment.

## Introduction

Dementia, defined as a collection of clinical syndromes including impairment of memory, judgment, reasoning, and changes in mood, behaviors, and communication abilities, is one of the most disabling conditions affecting the aged population (Kawas and Corrada 2006). Vascular dementia (VD), also known as vascular cognitive impairment (VCI), results from brain damage due to inadequate blood supply and is the second most common form of dementia only after Alzheimer's disease (AD). VD exhibits as a range of subtypes depending on the pathophysiology of the vascular diseases (Moretti et al. 2005). Vascular dementia induced by subcortical ischemic vascular disease (SIVD) has been proposed as a highly prevalent and homogeneous subtype of VD, and thought to be caused by small vessel infarct or ischemia occurring within the basal ganglia, thalamus, and white matter, leading to subcortical vascular injury (Moorhouse and Rockwood 2008). According to clinical manifestations, subcortical ischemic vascular cognitive impairment without dementia and subcortical vascular mild cognitive impairment (SVMCI) are termed prodromal stages of SIVD. Individuals at these stages exhibit evidence of relevant vascular risk factors, such as cerebrovascular disease with lower severity and no dementia, or a moderate level of cognitive impairment which does not fulfill diagnostic criteria for dementia (Galluzzi et al. 2005). The mild cognitive decline features include relative sparing of memory, dysexecutive syndrome, gait disorders, extrapyramidal signs, and other behavioral and psychological symptoms (Galluzzi et al. 2005). These conditions are more prevalent in clinic, and the high risk of further progression to dementia makes these conditions target for therapeutic interventions to slow or prevent the progression, through risk factors management and appropriate treatment (Seo et al. 2010).

In last two decades, thyroid status has emerged as a potential independent risk factor for the development of reversible cognitive impairment (Yoshimasu et al. 1991; Ganguli et al. 1996). Thyroid hormones have been demonstrated to play central roles in the development of central neural system, maintenance of normal neural functions, as well as the regulation of protein, fat, and carbohydrate metabolism (Ichibangase et al. 1990). The correlation between thyroid functional state and the risk of AD and VD has been widely investigated, by assessing the serum levels of thyroid hormone including triiodotyron (T3), thyroxin (T4), and thyroid‐stimulating hormone (TSH) in aged population (Dobert et al. 2003; Forti et al. 2012). One of the biological mechanisms underlying the relationship between thyroid dysfunction and dementia is suggested to be associated with cardiac vascular disease, which can contribute to cognitive impairment in later life (Wijsman et al. 2013). A number of cross‐sectional studies addressed the high prevalence of subclinical thyroid disease, in dementia patients with normal free thyroxin (FT4) and higher or lower TSH (Roberts et al. 2006; Ceresini et al. 2009). Both subclinical hyper‐ and hypothyroidism have been established as risk factors for cognitive impairment (Luboshitzky et al. 2002; Ceresini et al. 2009; Bensenor et al. 2010; Agarwal et al. 2013). Positive associations of both T3 and T4 with cognitive performance have been indicated in some studies, whereas other studies failed to demonstrate the same outcome (Prinz et al. 1999; Iervasi et al. 2003). Furthermore, autoimmune thyroid disease was associated with an increased risk of dementia by assessing the levels of antibody to thyroid peroxidase (TPO‐Ab) and thyroglobulin antibody (TG‐Ab) in subjects (Kalmijn et al. 2000). Although the findings are inconsistent, possibly due to the clinically heterogeneous conditions of dementia, the serum levels of thyroid hormones still hold potential to be potent markers to predict and evaluate cognitive decline in aged population. Nevertheless, investigation into association of thyroid dysfunction with a vascular dementia subtype SIVD and its predementia conditions is sparse.

In this study, we focused on the investigation of elderly cohorts with highly homogeneous dementia SIVD. We observed the clinical differentiation among normal controls and patients with different extent of cognitive decline. We aimed to reveal the association between thyroid dysfunction and cognitive impairment in SIVD, and determine whether thyroid hormones were able to predict the risk of developing SVMCI and VD. The correlation of autoimmune thyroid disease to SIVD was also explored.

## Materials and Methods

### Participants

A total of 161 patients diagnosed with SIVD aged from 54 to 75 were recruited from the Department of Neurology in The First Affiliated Hospital of Anhui Medical University and Tongling Forth People's Hospital from February 2013 to August 2014. Fifty‐four healthy participants (25 men, 29 women; mean age: 65 ± 9 years) were recruited from the Health Examination Center of Tongling Forth People's Hospital as controls. All control participants were functioning normally and had no sign of cognitive decline, without prior or present neurological or psychiatric disorders.

Each enrolled subject underwent a standardized personal interview for acquirement of data on basic information, lifestyle, clinical diagnosis, and medical history, and permitted an extensive clinical assessment and the collection of blood samples for the determination of serum biomarkers. Written informed consent was given by all participants. Health status of controls and patients was recorded by assessing vascular risk factors including hypertension, diabetes, history of cardiovascular disease and stroke, and long‐term past or present smoking (≥10 years) (Table 1). Hypertension was defined if the subject presented a systolic blood pressure >140 mm Hg and/or diastolic blood pressure >90 mm Hg. Diabetes mellitus was defined if the subject presented a fasting blood glucose ≥126 mg/dL (Bensenor et al. 2010).

**Table 1:** General characteristics of study population

Characteristics	Disease status			
	Control (*n* = 54)	SIVD		
		SIVDND (*n* = 52)	SVMCI (*n* = 55)	VD (*n* = 54)
Age (year)	65 ± 9	64 ± 10	66 ± 9	65 ± 10
Gender (Male/Female)	25/29	25/27	28/27	27/27
Weight (kg)	69 ± 12	70 ± 10	71 ± 11	69 ± 13
Education (≤3 years)	18 (33.3%)	19 (36.5%)	17 (30.9%)	19 (35.2%)
Hypertension	44 (81.5%)	41 (78.8%)	45 (81.8%)	43 (79.6%)
Diabetes mellitus	6 (11.1%)	6 (11.5%)	7 (12.7%)	5 (9.3%)
History of cardiovascular disease	8 (14.8%)	9 (17.3%)	8 (14.5%)	9 (16.7%)
History of stroke	1 (1.85%)	1 (1.92%)	2 (3.6%)	2 (3.7%)
Smoking (≥10 years)	16 (29.6%)	18 (34.6%)	17 (30.9%)	18 (33.3%)

Data are presented as means ± SD or numbers with percentages in parentheses.

SIVD, subcortical ischemic vascular disease; SIVDND, subcortical ischemic vascular disease no dementia; SVMCI, small vascular mild cognitive impairment; VD, vascular dementia.

Exclusion criteria for controls and all patients were: suspicion of Alzheimer's disease, diagnosis with a chronic or degenerative diseases, known thyroid disease, history of brain injury or alcohol abuse, treatment with medication which could influence thyroid or brain function within 4 weeks before the testing, inability to give an informed consent, and unclassifiable cognitive status because of inability to complete the psychological test due to aphasia, intelligence disturbance, depression, and psychiatric disorders. Specifically, SIVD diagnosis were determined in accordance with internationally accepted diagnostic criteria (Roman et al. 2002), and AD participants were excluded, based on the diagnosis at a multidisciplinary case conference using the National Institute of Neurological and Communicative Disorders and Stroke (NIHCDS) and the Alzheimer's Disease and Related Disorders Association (ADRDA) diagnostic criteria.

### Assessment of dementia

Following the criteria made by Erkinjuntti et al. (2000) and Winblad et al. (2004) coupled with the evidence on functional magnetic resonance imaging (fMRI) of subcortical vascular lesions of moderate to severe levels of either white matter lesion (MWL) or lacunar infarct (LI), the patients were classified at the time of recruitment into three groups based on severity of cognitive impairment: 52 patients with no dementia (SIVDND; 25 men, 27 women; mean age: 64 ± 10 years), 55 patients with mild cognitive impairment (SVMCI; 28 men, 27 women; mean age: 66 ± 9 years) and 54 patients with VD (27 men, 27 women; mean age: 65 ± 10 years). The numbers of patients with SIVD‐MWL and SIVD‐LI in each group were found to be identical. Formal neuropsychological tests were performed by a trained neuropsychological technician for all participants, including Mini‐Mental State Examination (MMSE), Montreal Cognitive Assessment (MoCA), Clinical Dementia Rating (CDR), Activities of Daily Living Scale (ADL), and Instrumental Activities of Daily Living (IADL). The ADL and IADL referred to daily living activities and general functional status in a dependent or independent lifestyle. The MMSE, MoCA, and CDR were quantitative screening instruments applied to evaluate global cognitive performance in memory, attention, language, recall, orientation, and executive functions. Dementia severity was assessed by MMSE and MoCA scores in a range from 10 to 30. Patients with CDR score greater than 2.5 were excluded.

### Blood thyroid hormone

Venous blood sample of 3 mL for each subject was drawn at 7 am following a 12‐h fast. The serum was collected after centrifugation within 30 min of specimen collection and stored at −40°C for hormone and biochemistry measurements. Serum thyroid‐stimulating hormone (TSH), total triiodothyronine (TT3), free triiodothyronine (FT3), total thyroxine (TT4), and free thyroxine (FT4) were measured with radioimmunoassay (normal reference interval in our laboratory of each: TT3: 0.9–2.8 nmol/L; FT3: 3.5–8.3 pmol/L; TT4: 58–161 nmol/L; FT4: 10–28 pmol/L; TSH: 0.4–4 mIU/L). Thyroid state was determined by evaluating the measured parameters. Euthyroidism was defined as a normal serum level of TSH. Thyroglobulin antibody (TG‐Abs) (normal < 116 kIU/L) and antithyroid peroxidase antibody (TPO‐Abs) (normal < 50 kIU/L) were quantified with ELISA (Milenia, DPC, Los Angeles, CA). Positive outcomes for antibodies could indicate autoimmune thyroid problems.

### Statistical analysis

Data were expressed as mean ± standard deviation or numbers with percentages in parentheses. All statistical analysis was performed with program SPSS 15.0 (SPSS Inc., Chicago, IL). A logistic regression model was established to analyze association between dementia status (SIVDND, SVMCI, VD, and Control) and thyroid parameters (TT3, FT3, TT4, FT4, TSH, TG‐Ab, and TPO‐Ab). The thyroid parameter was considered as dependent variable and the disease status was independent variable. In the same group of each dementia status, one‐way analysis of variance followed by a Tukey post hoc test was used to analyze data of variables. Significant statistical differences were considered to be present at *P *<* *0.05.

## Results

The general characteristics of the study participants per dementia status are listed in Table 1, and all four groups had a similar profile. In each group, gender was almost evenly distributed. There were no significant differences in age, gender, weight, and education level among the four groups. A high prevalence of hypertension that might be associated with thyroid dysfunction was revealed in both patient and healthy control groups, but the rate of hypertension between the groups were similar (around 80% for each group). There were no significant differences in prevalence of diabetes, history of cardiovascular disease and stroke, and smokers among the groups, indicating the health status of each group was also similar.

All 215 participants of the study underwent a number of cognitive and functional performance tests, and the neuropsychological scores for each group were reported in Table 2. All four groups had a similar score on ADL and IADL. Participants with SVMCI had a dramatically lower scores of MMSE (mean 20.6 ± 4.9) and MoCA (mean 16.8 ± 2.5) than those of the control group (mean MMSE 28.3 ± 4.4, mean MoCA 27.3 ± 2.4; *P* < 0.01). The VD group showed a further significant decline in both the MMSE and MoCA scores compared to SVMCI (*P* < 0.05), suggesting higher scores leading to worse functional performance. The SIVDND patients showed better performance. No significant statistical difference was found between SIVDND and control groups, although there was a slight decrease of the scores in SIVDND group (mean MMSE 26.8 ± 4.0, mean MoCA 25.5 ± 2.5) compared to the controls (mean MMSE 28.3 ± 4.4, mean MoCA 27.3 ± 2.4). Likewise, a significant difference in CDR scores was found among the SVMCI, VD, and control groups. The mean CDR scores were elevated with increased severity of the disease and reached up to 1.3 ± 0.4 in the SVMCI group and 2.1 ± 0.8 in the VD group, compared with only 0.2 ± 0.1 in the SIVDND group and control group which was considered practically to be 0. Furthermore, white matter hyperintensity (WMH) volume was determined. The WMH volume was decreased in SIVD groups when compared to control (35.7 ± 3.1 cm^3^), especially in SVMCI and VD groups (32.7 ± 2.6 and 30.1 ± 2.8 cm^3^, respectively).

**Table 2:** Various cognitive and functional performance tests of SIVD patients and control subjects

Test	Disease status			
	Control (*n* = 54)	SIVD		
		SIVDND (*n* = 52)	SVMCI (*n* = 55)	VD (*n* = 54)
ADL	4.1 ± 1.4	4.0 ± 1.3	4.2 ± 1.3	4.1 ± 1.5
IADL	5.6 ± 1.3	5.5 ± 1.6	5.8 ± 1.7	5.5 ± 1.5
MMSE	28.3 ± 4.4	26.8 ± 4.0	20.6 ± 4.9^a^	16.4 ± 4.8^a,b^
MoCA	27.3 ± 2.4	25.5 ± 2.5	16.8 ± 2.5^a^	10.7 ± 2.6^a,b^
CDR	–	0.2 ± 0.1	1.3 ± 0.4^a^	2.1 ± 0.8^a,b^
WMH volume (cm^3^)	35.7 ± 3.1	33.9 ± 2.9	32.7 ± 2.6^a^	30.1 ± 2.8^a,b^

Data are presented as means ± SD. ^a^ *P* < 0.01 vs. control; ^b^ *P* < 0.05 vs. SVMCI.

SIVD, subcortical ischemic vascular disease; ND, subcortical ischemic vascular disease no dementia; SVMCI, small vascular mild cognitive impairment; VD, vascular dementia; ADL, activities of daily living scale; IADL, instrumental activities of daily living; MMSE, mini‐mental state examination; MoCA, montreal cognitive assessment; CDR, clinical dementia rating; WMH, white matter hyperintensity.

**Figure 1:**
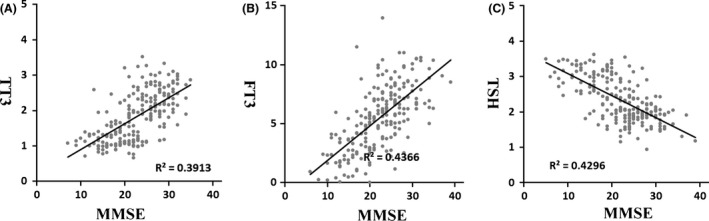
Correlations between MMSE scores and changes in the serum levels of (A) TT3, (B) FT3, and (C) TSH in all patients (*n* = 215).

The association between thyroid status and cognitive decline in SIVD patients was investigated by exploring the statistical difference among mean values of thyroid hormone levels in the four groups. As demonstrated in Table 3, there were highly significant correlations between SIVD status with both TT3 and FT3. Compared to the control group, TT3 and FT3 levels in SVMCI and VD groups were remarkably lower (*P* < 0.01). Notably, the mean value of FT3 level in VD group (2.34 ± 1.51 pmol/L) was decreased below the normal reference range (3.5–8.3 pmol/L). Moreover, a significant correlation between TSH level and SIVD group was also reported (*P* < 0.01). Elevated TSH values were reported for SVMCI (2.48 ± 0.44 mIU/L) and VD (3.07 ± 0.37 mIU/L) when compared with the control (1.87 ± 0.32 mIU/L), while all the levels were within the normal range (0.4–4 mIU/L). With further analysis between SVMCI and VD groups, lower T3 levels and higher TSH values were recorded in VD group compared to SVMCI group, and all differences were considered to be significant (*P* < 0.05). Combining the thyroid status with the cognitive impairment evaluation, as expected, the TT3 and FT3 serum levels presented positive linear correlations to MMSE scores, whereas negatively correlated to TSH levels (Fig. 1). For SIVDND group, both TSH and T3 values were observed corresponding to be slightly altered as compared to the control, but the differences were not significant. The same statistical analysis was performed for T4, but no significant association was found for both TT4 and FT4 among the four groups. In addition, immune complexes TPO‐Ab and TG‐Ab were detected in our study and no significant difference was identified between SIVD patients and healthy controls. Worth of mention, MMSE scores were positively correlated with WMH volume (*R* = 0.54).

**Table 3:** Thyroid function tests of SIVD patients and control subjects

Parameters	Disease status			
	Control (*n* = 54)	SIVD		
		SIVDND (*n* = 52)	SVMCI (*n* = 55)	VD (*n* = 54)
TT3 (nmol/L)	2.46 ± 0.45	2.32 ± 0.48	1.48 ± 0.39^a^	1.08 ± 0.21^a,b^
TT4 (nmol/L)	82.34 ± 13.53	83.77 ± 14.13	85.12 ± 13.88	90.27 ± 17.44
FT3 (pmol/L)	7.82 ± 1.79	7.46 ± 1.92	4.78 ± 1.34^a^	2.34 ± 1.51^a,b^
FT4 (pmol/L)	26.55 ± 2.71	27.34 ± 2.81	24.07 ± 2.42	23.11 ± 2.49
TSH (mIU/L)	1.87 ± 0.32	1.95 ± 0.35	2.48 ± 0.44^a^	3.07 ± 0.37^a,b^
TG‐Ab (kIU/L)	85.56 ± 16.21	82.37 ± 15.37	89.19 ± 17.48	90.27 ± 18.22
TPO‐Ab (kIU/L)	25.69 ± 4.17	23.63 ± 4.30	27.24 ± 5.11	26.18 ± 5.32

Data are presented as means ± SD. ^a^ *P* < 0.01 vs. control; ^b^ *P* < 0.05 vs. SVMCI.

SIVD, subcortical ischemic vascular disease; ND, subcortical ischemic vascular disease no dementia; SVMCI, small vascular mild cognitive impairment; VD, vascular dementia.

## Discussion

To the best of our knowledge, this is the first study to investigate the relationship among thyroid status, cognitive status, and severity of SIVD. A large number of previous literatures have implicated thyroid dysfunction as a risk factor for AD and VD, as the critical roles of thyroid hormones in the maturation of the neural system and maintenance of normal neural function were well established. However, the association between thyroid disorder and cognitive decline still remains controversial, due to the inconsistent results in many previous cross‐sectional studies. Both subclinical hyper‐ and hypothyroidism, due to abnormal TSH level, have been demonstrated to increase the risk of AD and VD in previous study, but other studies failed to demonstrate the same results (Tan et al. 2008). These conflicted results may arise from the variety of sample population, age, size, weak power of follow‐up study, and possible positive protective effect of TSH on preventing cognitive decline (Mungas et al. 2001).

There are a limited number of studies applied to assess the correlation of thyroid dysfunction with any subtype of VD and its cognitive features. Our study focused on the investigation within SIVD cohorts, and determined their dementia state according to the evidence on clinical symptoms and detailed neuropsychological tests on multiple cognitive domains. SVMCI is a highly prevalent condition in SIVD population, and is thought to be a reversible transition stage to vascular dementia. It has been identified as a preventive and therapeutic target in clinic aimed to reduce disability burden in the elderly (Poggesi et al. 2012). SVMCI and VD patients with worse cognitive performance presented distinctly lower MMSE and MoCA scores, and higher CDR scores than the healthy control, whereas no significant difference was found between SIVDND group and the control. The daily activities and functional status were similar between SVMCI and severe VD patients in this study. However, the cognitive performance of VD patients was significantly declined, as indicated by the significantly altered scores of several neuropsychological tests MMSE, MoCA, and CDR, all as measurements of global cognitive function. Our findings indicated the examination scores of SVMCI with better performance was significantly distinguished from those of VD, which suggested the potential of the neuropsychological profile employed as an instrumental determinant to isolate the predementia state SVMCI from SIVD. Performing the functional examination coupled with the conventional factors to recognize SVMCI individuals would allow physicians to optimize the diagnostic criteria of SVMCI and VD, and to relate the cognitive dysfunction to functional lesion status and the thyroid biomarkers, therefore contribute to the understanding of the progression mechanisms, predicting the risk of progression, and devising appropriate interventions to prevent the onset (Frisoni et al. 2002).

The mechanism of the association between thyroid dysfunction and cognitive impairment is still poorly understood. One possible explanation is that the correlation between thyroid disorder and cerebrovascular risk can further contribute to dementia development. Our study has implicated thyroid status in cognitive impairment of SIVD independent of sociodemographic and vascular risk factors. A number of previous literatures proposed thyroid hormones as risk predictors of dementia. Subclinical hypothyroidism with T4 and elevated TSH has been identified as a common predisposing factor of depression, cognitive impairment, and dementia (Davis et al. 2003). Our study indicated a consistent positive correlation between TSH levels and cognitive decline in SIVD. However, most of the associations found in subclinical hypothyroidism concerned participants in particular with a TSH level of 10 mIU/L or above (Roman et al. 2002), and our study indicated there was a significant association between higher TSH to cognitive decline in SIVD, even with the TSH concentration within the normal reference range. By a number of clinical research, the prevalence of thyroid dysfunction has been demonstrated following cerebral infarction, and the process of dementia development may lead to a reduction in secretion of thyroid hormones resulting in neurodegeneration (Tan and Vasan 2009). Furthermore, the direct effects of thyroid hormone deficiency were also investigated, where some studies suggested altered thyroid hormone levels may result in impaired regulation of gene expression of cerebral amyloid‐*β* proteins that may contribute to the development of AD (Tan and Vasan 2009). Elevated TSH levels were capable of leading to increased oxidative stress and neuronal death, which may explain our findings (Fu et al. 2012). Therefore, the altered TSH levels could be the consequence or cause of cognitive decline in SIVD (Tan and Vasan 2009). Our results are consistent with (Forti et al. 2012), which suggested that high TSH level was associated with increased risk of VD. It has been reported that autoimmune thyroid disorders may be the cause of increased serum TSH highly prevalent among AD patients (Biondi and Cooper 2008). To validate this speculation, serum thyroid autoantibodies including TPO‐Abs and TG‐Abs were measured in this study. However, no significant difference of serum TPO‐Abs and TG‐Abs were found between SIVD patients and the control, indicating the increased TSH did not act as the marker of autoimmunity.

This study found that cognitive function was closely related with serum T3, which has been widely characterized to be a notable preventive and therapeutic target in thyroid disorders, and even in neuropsychical diseases (Scherr et al. 2014; Lin et al. 2015). Serum free T3 exhibited a remarkably decrease with cognitive decline of SIVD even below the normal range in VD group, suggesting its potential as indicator reflecting thyroid and cognitive status in SIVD patients. A consistent association was also found for TT3. A linear relationship between thyroid hormone and MMSE scores was conducted. Higher TSH and lower T3 significantly lowered the MMSE scores, which indicated worse cognitive performance. A central role of T3 in neuronal differentiation has been uncovered, which directed stem cells to generate clones of neuro‐oligodendrocytes from the central nervous system (Johe et al. 1996). Previous study manifested that decreased serum FT3 could interfere with the normal differentiation of astrocytes by down‐regulating the expression of neurotransmitter *β*‐adrenergic receptors in the cells and the release of a number of neuro‐active compounds (Das and Paul 1994). In some animal studies, both short‐term and long‐term drug treatments with thyroid hormones have exhibited effects on reduction of the cognitive impairing process. The treated animals presented improved spatial cognitive performance and enhanced cholinergic activity in frontal cortex and hippocampus (Smith et al. 2002). An alternative explanation may be attributed to the critical roles of T3 in the maintenance of inner mitochondrial membrane fluidity and permeability through regulating the fatty acid and phospholipid composition of the membrane. It is likely that T3 deficiency may lead to the onset of mitochondrial dysfunctions, thus affecting normal physiological processes including brain metabolic activity and intracellular signal transduction, and contributing to the pathophysiological processes such as cerebral ischemia damage (Shindo et al. 2016). Hence, thyroid hormone drug intervention with T3 is noteworthy as a therapeutic target at the early stage of SIVD to improve the cognitive performance of patients and restrict the dementia progression. Some studies also considered serum T4 as a major factor affecting cognitive performance and risk of processing dementia in the elderly (Prinz et al. 1999; Hogervorst et al. 2008). A previous study reported an association between high FT4 levels and an accelerated cognitive decline as well as dementia progression (Hogervorst et al. 2008). However, a significant association between T4 and dementia for SIVD was not discovered in this study, which is likely due to the small size of the cohort studied and the lack of follow‐up data. But, T4 can still be a potential research and clinical target on diagnosis and therapy of dementia involving thyroid dysfunction and other risk factors.

To make our study highly appropriate to detect a possible association between thyroid dysfunction and SIVD development, a significant strength in our study design is the restricted homogeneous dementia nature of the participants, strict exclusion criteria of participants with risk factors, extensive diagnostic classification for SIVDND, SVMCI, and VD, and clinical and neuropsychological verification of the participants. Several limitations of our study, however, are the relatively small sample size, and the lack of repeated measurement for thyroid hormones and follow‐up data, that may have affected the precision.

In conclusion, we found higher serum TSH and lower T3 levels may be associated with cognitive impairment induced by SIVD. Our study recognized the neuropsychological features of VD stages, and proposed prospective clinical implication of the early dementia stage on the treatment of reversible VD with thyroid dysfunction. Further studies with follow‐up data and large sample size are necessary to clarify the mechanisms behind this relationship.

## Conflicts of Interest

The authors declare no conflict of interest.
